# Supplementary material for: Identification of photoperiod-induced specific miRNAs in the adrenal glands of Sunite sheep (Ovis aries)
Source: Front Vet Sci. 2022 Jul 22;9:888207. doi: 10.3389/fvets.2022.888207 (PMC9354845; doi:10.3389/fvets.2022.888207)
Supplement: Supplementary file 1 [file Data_Sheet_1.ZIP › Supplementary file/miRDeep2 file.zip!/miRDeep2 file/Novel_Structure/LP42A2/Novel_655.pdf]

Provisional ID	:	NC_019480.2_33707
Score total	:	0.5
Score for star read(s)	:	-1.3
Score for read counts	:	0
Score for mfe	:	0.2
Score for randfold	:	1.6
Score for cons. seed	:	
Total read count	:	13
Mature read count	:	13
Loop read count	:	0
Star read count	:	0

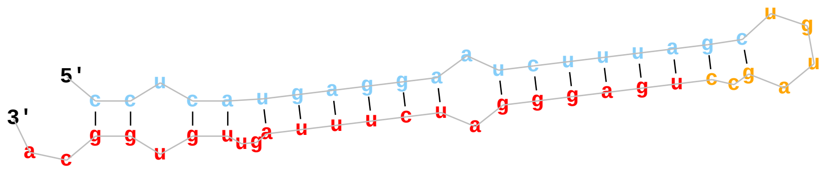

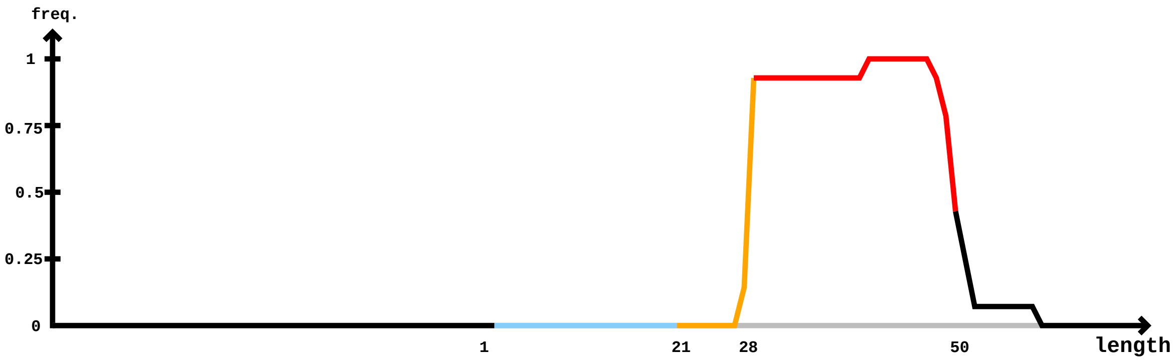

Star Mature

```
5'- uaaauauucuaauuuauuuauuugacuguguugggucuuaguugccucaugaggaaucuuuagcuguagccugagggaucuuuaguuguggcaugugggaucuaguucccug   -3'    exp
    ((((((........))))))..........((((((((((..((((.((((((((.((((((((....).))))))).))))))..)).))))..))))))))))......        reads      mm        sample
    ......................................................................cugagggaucuuuaguuguggc....................        2          0         seq
    .......................................................................ugUgggaucuuuaguugug......................        1          1         seq
    .......................................................................ugUgggaucuuuaguugugg.....................        2          1         seq
    .......................................................................ugUgggaucuuuaguuguggc....................        2          1         seq
    .......................................................................ugagggaucuuuaguuguggU....................        1          1         seq
    .......................................................................ugUgggaucuuuaguuguggca...................        5          1         seq
    ...................................................................................aguuguggcaugugggau...........        1          0         seq
```
